# Extensive Spiculated Follicular Porokeratosis With Alopecia: A Case Report

**DOI:** 10.1002/ccr3.70100

**Published:** 2025-01-10

**Authors:** Mitra Abdolvand, Roya Radanfar, Mozhdeh Sepaskhah, Fatemeh Sari Aslani, Mojgan Akbarzadeh Jahromi

**Affiliations:** ^1^ Department of Dermatology, School of Medicine Shiraz University of Medical Sciences Shiraz Iran; ^2^ Molecular Dermatology Research Center, Department of Dermatology, School of Medicine Shiraz University of Medical Sciences Shiraz Iran; ^3^ Molecular Dermatology Research Center, Department of Pathology, School of Medicine Shiraz University of Medical Sciences Shiraz Iran; ^4^ Department of Pathology, School of Medicine Shiraz University of Medical Sciences Shiraz Iran

**Keywords:** alopecia, case reports, dermoscopy, pathology, porokeratosis, scalp

## Abstract

Follicular porokeratosis (FPK) is a rare subtype of porokeratosis. Follicular porokeratosis mainly occurs in men and may be localized or diffuse. Involvement of the scalp is rarely reported, and we found only one case of alopecia due to scalp FPK. Diabetes mellitus has not been reported in association with FPK We report a 35‐year‐old diabetic woman that presented with brown follicular keratotic papules and spicules involving the scalp, face, trunk, and extremities, accompanied by scalp alopecia. The differential diagnoses were lichen planopilaris, papular eczema, and phrynoderma. Histopathologic examination of skin punch biopsies revealed the follicle‐centered cornoid lamella as the feature of FPK. The patient was treated with oral isotretinoin, and the lesions improved partially within 6 weeks of follow‐up. Follicular porokeratosis might be considered among the differential diagnoses of keratotic papules (with or without spicules) and in the cases of alopecia with keratotic papules. Diabetes mellitus may be associated with FPK.

**Summary**

- Follicular porokeratosis should be considered among the differential diagnoses of keratotic papules (with or without spicules) and in cases of alopecia with keratotic papules.
- Dermoscopy could be helpful in the diagnosis of FPK.
- Diabetes mellitus type 2 may be associated with FPK.

## 1 Introduction

Porokeratosis (PK) is an epidermal disorder of keratinization caused by mevalonate pathway abnormalities [1]. Different clinical subtypes of PK are described. The common types include disseminated superficial actinic porokeratosis, porokeratosis of Mibelli, and linear porokeratosis. Disseminated superficial porokeratosis, eruptive disseminated porokeratosis, porokeratosis palmaris et plantaris disseminate, punctate porokeratosis, porokeratosis ptychotropica, penoscrotal porokeratosis, and follicular porokeratosis (FPK) are the less common types of PK [2]. The clinical appearance, predilection site, and symptoms vary in various forms of PK [2]. Cornoid lamella is the common histopathologic feature in all types of PK [1, 2].

Follicular porokeratosis is a rare form of PK, characterized by the cornoid lamella filling the hair follicle infundibulum [3]. Follicular porokeratosis may be isolated, or associated with other PK forms [4, 5]. Most of the FPK cases present with small number of lesions [3, 6, 7, 8, 9], although few cases with diffusely distributed lesions have been reported [5, 10]. Follicular porokeratosis rarely involves the scalp, leading to alopecia, with only one case reported previously [3, 5, 11]. Porokeratosis, but not FPK, has been reported in association with diabetes mellitus type 2 [12, 13].

Herein, we report a case of extensive FPK with spicules that caused alopecia of the scalp hair in a diabetic woman.

## 2 Case History/Examination/Presentation

A 35‐year‐old woman presented to the dermatology clinic to manage generalized brownish keratotic papules and disseminated follicular spicules. She was recently diagnosed with diabetes mellitus (DM) type 2.

The lesions initially appeared on the upper limbs 2 years ago and gradually extended to the lower limbs, chest, back, buttocks, face, and scalp, leading to alopecia in the last year.

**FIGURE 1:**
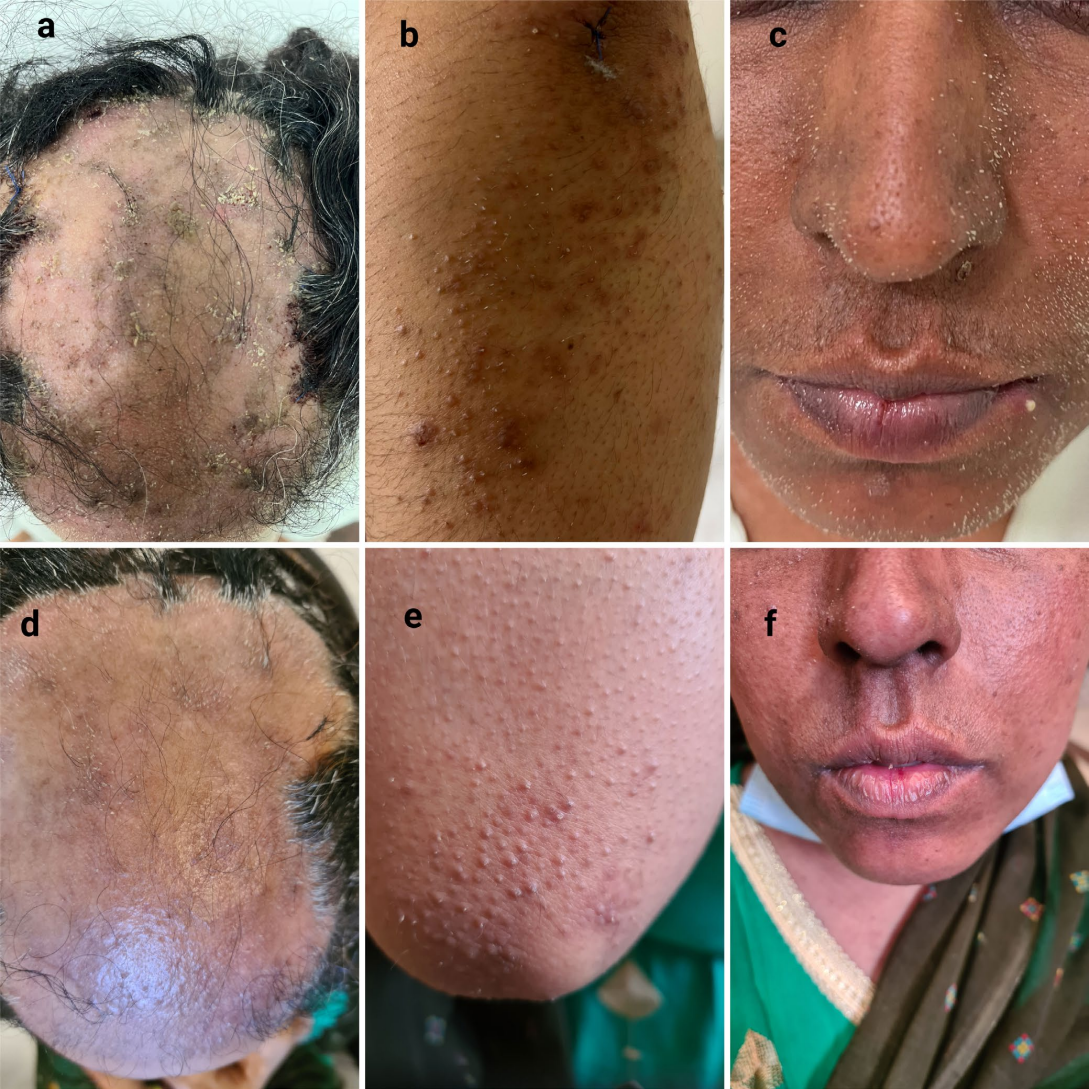
Clinical manifestations of patient. (a–c) Before treatment. (a) Alopecia with brown keratotic papules and follicular spicules over the frontal and vertex regions. (b) Brown keratotic papules and follicular spicules over the elbow. (c) Keratotic spicules over the face. d‐f. After six weeks treatment with isotretinoin. Improved lesions on the scalp (d), elbow (e), and face (f).

Physical examination of the scalp showed large areas of alopecia with keratotic papules and follicular spicules over the frontal and vertex regions. Disseminated brown follicular papules with spicules measuring 1–2 mm were observed over the face, trunk, and upper and lower limbs (Figure 1) and were associated with folliculitis, xerosis, and mild pruritus. The patient had lesions in both sun‐protected and sun‐exposed areas of the body. The nails and mucous membranes were normal. She had no history of other diseases (except DM type 2), radiation, or drug allergy. No similar lesions were noted in the other family members. The results of laboratory tests were in normal range (except for fasting blood sugar: 160 mg/dL [normal range: 70–100 mg/dL] and hemoglobin A1c: 8.9% [normal: < 6.0%, prediabetes: 6.0%–6.4%, diabetes: > 6.5%]).

## 3 Methods (Differential Diagnosis, Investigations, and Treatment)

The clinical differential diagnoses included lichen planopilaris, papular eczema, and phrynoderma. Skin biopsies were performed on the buttock and scalp lesions.

**FIGURE 2:**
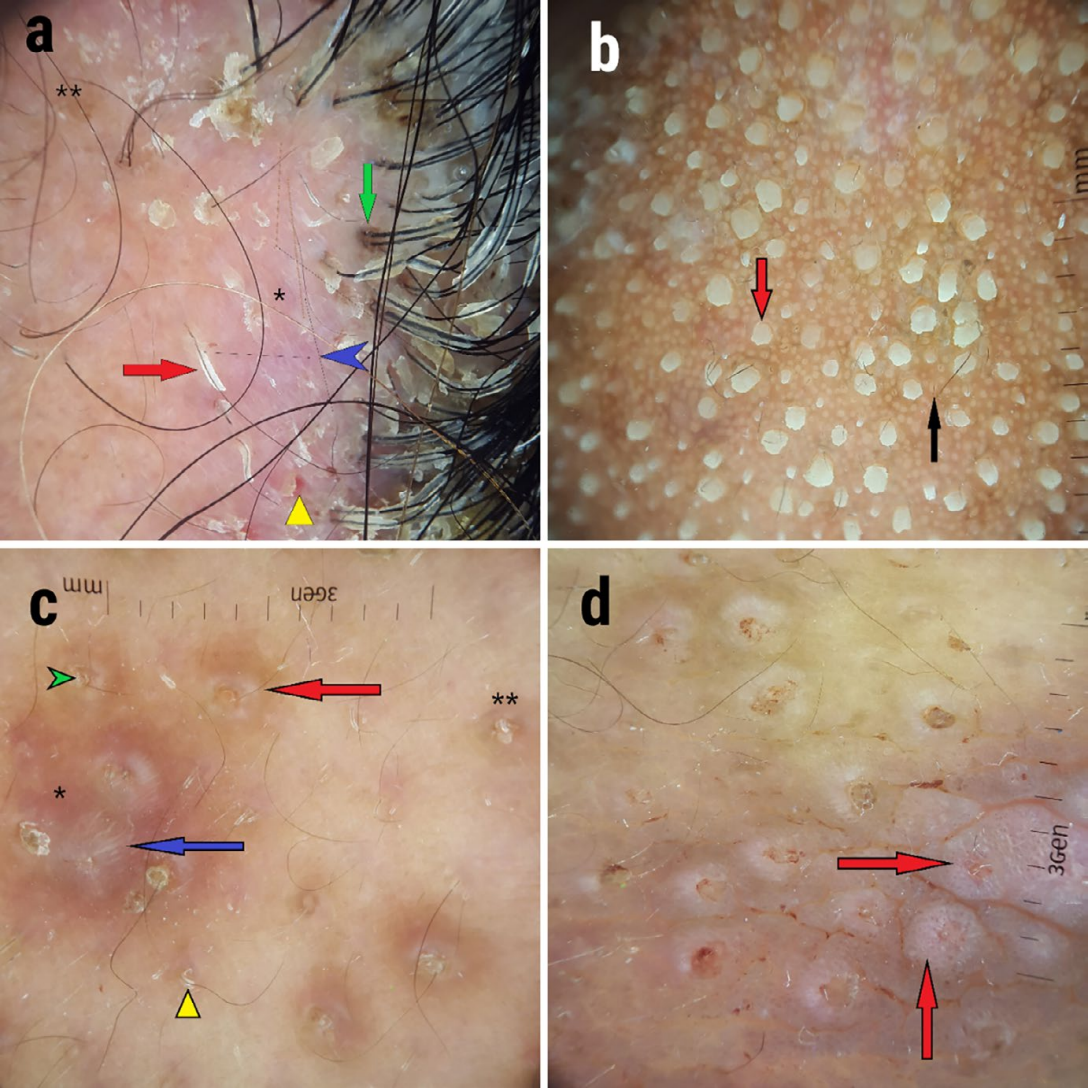
Dermoscopic features of follicular porokeratosis lesions. (a) Scalp. Erythematous background (asterisk), scales, white linear structures (blue arrowhead), gray‐brown pigmentation (double asterisk), gray‐brown globules in the dilated hair openings (green arrow), blood spots (yellow triangle), and keratin casts protruding around hair follicles (red arrow). (b) Nose. Peripheral white rims around hair openings (black arrow) and thick keratin spicules (red arrow). (c) Buttock. Erythematous areas (asterisk), gray‐brown pigmentation (double asterisk), peripheral white rims (red arrow) and radial white lines around hair openings (blue arrow), yellow‐white follicular keratotic plugs (green arrowhead), and keratin casts protruding around hair follicles (yellow triangle). (d) Buttock. Dotted vessels (red arrows) are prominent in some lesions.

Dermoscopy of scalp lesions showed erythematous background, scales, white linear structures, gray‐brown pigmentation, gray‐brown globules in the dilated hair openings, blood spots, and keratin casts protruding around hair follicles (Figure 2a). Peripheral white rims around hair openings and thick keratin spicules were the findings of the nose dermoscopy (Figure 2b). Dermoscopy of buttock lesions showed erythematous areas and gray‐brown pigmentation. Peripheral white rims and radial white lines around hair openings were evident. Also, there were yellow‐white follicular keratotic plugs, keratin casts protruding around hair follicles, and dotted vessels (Figure 2c,d).

**FIGURE 3:**
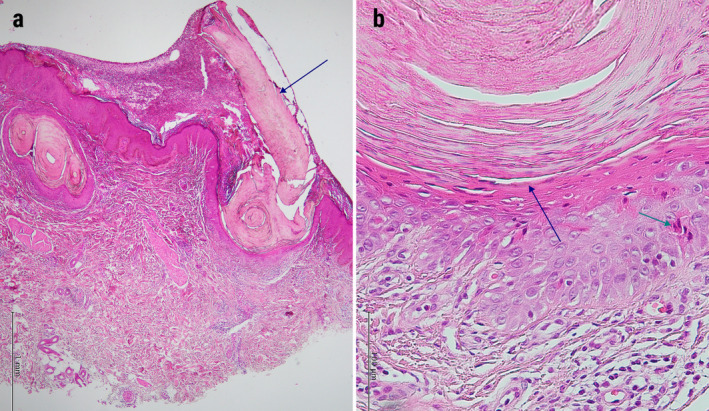
Histopathologic features of buttock follicular porokeratosis lesions. (a) Follicle‐centered parakeratotic columns, acanthosis, and absence of underlying granular layer with a few scattered dyskeratotic keratinocytes (cornoid lamella) (blue arrow). H&E stain. 40× (b) Parakeratotic column, absence of underlying granular layer (blue arrow) with some scattered dyskeratotic keratinocytes (green arrow) (cornoid lamella) H&E stain. 400×.

**FIGURE 4:**
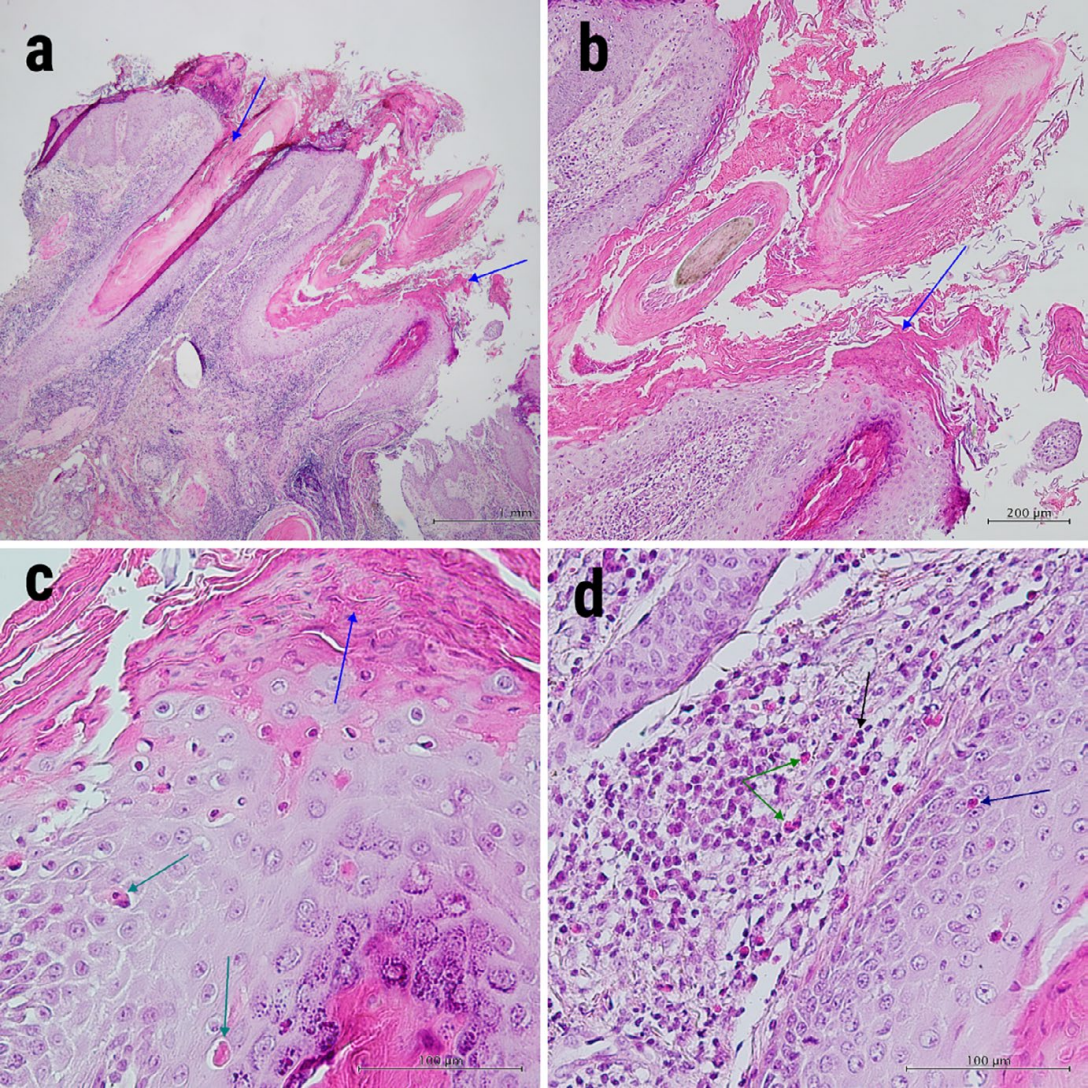
Histopathologic features of scalp follicular porokeratosis lesions. (a and b) Follicle‐centered parakeratotic columns (blue arrows), absence of underlying granular layer with scattered dyskeratotic keratinocytes (cornoid lamella), and perifollicular inflammation. H&E stain. 40× (c) Parakeratotic (blue arrow) and prominent scattered dyskeratotic (green arrows) keratinocytes H&E stain. 100× (d) dermal perifollicular infiltration of lymphocytes (black arrow) and eosinophils (green arrows) with eosinophilic exocytosis (blue arrow) to follicular infundibulum. H&E stain. 400×.

Histopathological examination revealed hyperkeratosis, parakeratosis, dilated infundibula filled by ortho‐ and parakeratotic layers, acanthosis and absence of underlying granular layer. Also, a few scattered dyskeratotic keratinocytes and exocytosis of eosinophils were present in the follicular infundibulum. There were superficial and deep dermal perivascular and perifollicular dense lymphocyte and eosinophil infiltration (Figures 3 and 4). So, the diagnosis of follicular porokeratosis was confirmed. Oral isotretinoin 0.3 mg/kg/day was started, along with topical tacrolimus 0.01% twice daily.

## 4 Conclusion and Results (Outcome and Follow‐Up)

The treatment was well tolerated without adverse effects (except dry skin and lips), and the lesions partially improved in the 6 weeks follow‐up (Figure 1d–f), and the patient expressed her treatment satisfaction in the follow‐up. Despite treatment continuation, alopecia progressed, leading to extensive scarring alopecia in the next follow‐up.

The patient signed written informed consent regarding publishing their data and photographs. The researchers committed to maintaining patient confidentiality. The institutional ethics committee approved the case report (ethics code: IR.SUMS.MED.REC.1401.402).

## 5 Discussion

Porokeratosis, a rare skin disorder, comprises several clinical types, and all of these subtypes share the cornoid lamella in histopathology. Clinical and dermoscopic features of the reported cases of FPK are summarized in Table 1. Follicular porokeratosis has been reported more commonly in men, and only about one‐third of FPK patients are women. The age of reported cases ranged from 19 to 82 years [3, 17]. Our patient's age was within this age range.

**TABLE 1:** Clinical and dermoscopic features of the reported cases of follicular porokeratosis.

Study	Age/gender	Clinical features	Site	Alopecia	Pruritus	Associated disease(s)	Dermoscopy	Treatment	Other associated PK
de Almeida et al. [14]	43/F	Porokeratotic lesions with keratotic ridges	Arms and legs	No	NM	No	NM	NM	DSAP
	45/F	Desquamative plaques with follicular accentuation inside a hyperkeratotic edge	Back of the hand, ankle and arm	No	NM	No	NM	NM	PM
Yong et al. [15]	42/M	An erythematous, scaly plaque, with a raised, doubled‐edged border	Natal cleft	No	Yes	No	NM	5‐FU cream	PM
Pongpudpunth, Farber, and Mahalingam [16]	41/M	Multiple erythematous keratotic papules following a follicular distribution	Trunk and upper extremities	No	Yes	No	NM	High potency topical steroids and Plaquenil	No
Lee et al. [7]	26/F	Multiple, brown‐colored, ring‐like lesions without atrophic centers, and a half‐palm‐sized, arcuate‐shaped patch consisting of confluent brown‐colored, ring‐like lesions	Cheeks	No	No	No	NM	Topical pimecrolimus	No
Rocha‐Sousa et al. [17]	19/M	Multiple erythematous keratotic papules with a raised double‐edged border	Nose	No	No	No	NM	NM	No
Rifaioğlu et al. [8]	34/F	A 1 cm plaque, with hyperkeratotic ridge and depressed center	Nose	No	No	No	NM	NM	No
Kim et al. [6]	26/M	A 3 mm, circumferential, pale‐brown and smooth plaque	Nose	No	No	Cystic fibrosis, resected melanoma	NM	NM	No
	35/F	A 3 mm, round, pale‐yellow, scaly lesion	Nose	No	No	No	NM	NM	No
Zhao et al. [10]	35/M	Multiple sporadically distributed 1‐5 mm brownish papules	Gluteal folds	No	Yes	No	NM	NM	No
	50/M	Multiple erythematous plaques with a peripheral elevated keratotic ridge	Upper back and occipitonuchal area	No	Yes	No	NM	NM	No
	79/M	Multiple disseminated small black‐brown colored keratotic eruptions with elevated borders	Face and upper limbs	No	NM	No	NM	NM	No
Trikha et al. [18]	50/F	A 4 × 3 cm annular cluster of perifollicular keratotic papules each with a faint rim of erythema	Shoulder	No	Yes	No	NM	NM	No
Sud et al. [9]	69/F	An inflamed, increasingly pigmented lesion 7 mm in size	Neck	No	NM	No	NM	Curettage and cautery	No
	81/M	Multiple, discrete, discoid erythematous and brown macules and patches, with slight scale, and (some) atrophic	Trunk and limbs	No	No	BCC, Atypical fibroxanthoma	NM	Topical 5‐FU, 5% topical imiquimod, and topical mometasone furoate 0.1% ointment	No
	53/M	NM	Medial canthus	No	Yes	BCC	NM	NM	No
	56/M	Erythematous plaque	Forehead	No	NM	Gilbert syndrome, Gall stone	NM	NM	NO
Sun et al. [11]	66/M	Well‐defined lesions with brown periphery and several brown keratotic papules inside	Face, scalp, limbs, legs, arms, and torso	No	Yes	No	Annular scaly edge, atrophic center, and follicular keratotic papules	Cryotherapy	No
Tallon et al. [4]	42/M	Annular scaled lesion with a circumscribed margin and small keratotic papules	Sun‐exposed areas, predominantly on the forearms and lower legs	No	No	No	NM	NM	DSAP
Tokat et al. [19]	30/M	Multiple monomorphi, flat brownish papules with hints of peripheral and central hyperkeratosis	Legs	No	Yes	No	peripheral and central hyperkeratosis – occasionally with follicular accentuation – on a brownish, pale structureless background with focal linear vessels	topical and intralesional corticosteroids, 5‐FU cream	No
Young et al. [5]	26/M	Large geometric alopecia with follicular spicules, spicules at follicular orifices, follicular prominence with spicules	Scalp, nose, chins, ears, trunk, arms, lower legs	Yes	Yes	Sturge–Weber syndrome, Hidradenitis suppurativa	NM	Tretinoin cream, Amlactin cream, ketoconazole shampoo, acitretin	No
Gómez‐Zubiaur et al. [3]	82/M	Multiple pinkish macules, under 1 cm, with a slightly atrophic center and a serpiginous track	Scalp	No	Yes	No	Ill‐defined pinkish and violet areas with a prominent vascular network and yellow keratotic plugs in some follicles	NM	disseminated superficial PK
Bembry et al. [20]	42/F	Dusky hyperpigmented papules and nodules	Eyebrow, temples, forehead, and chin	No	NM	No	NM	Topical 5‐fluoracil, followed by cholesterol/lovastatin	No
Present case	35/F	Large areas of alopecia with keratotic papules and follicular spicules, Disseminated, 1–2 mm brown follicular papules with spicules	Scalp, face, trunk, and upper and lower limbs	Yes	Yes	Diabetes mellitus type 2, Folliculitis	Scalp: Erythematous background, scales, white linear structures, gray‐brown pigmentation, gray‐brown globules in the dilated hair openings, keratin casts protruding around hair follicles Nose: perifollicular white rims and thick keratin spicules, Buttock: radial white lines around hair opening, and dotted vessels	Oral isotretinoin 0.3 mg/kg/d Tacrolimus 0.1% ointment	No

Abbreviations: 5‐FU, 5‐fluorouracil; DSAP, Disseminated superficial actinic porokeratosis; F, female; M, male; NM, Not mentioned; PM, Porokeratosis of Mibelli.

The lesions are primarily erythematous or brown papules or plaques and brown follicular keratotic papules [1]. The presence of spiculated lesions and alopecia is very rare [5]. Our patient presented these rare clinical features. Follicular porokeratosis may occur in the context of other subtypes of PK [4, 14, 15] or as an independent presentation [3, 6, 7, 8, 16, 17]. Follicular porokeratosis lesions are usually asymptomatic, but some pruritic lesions (as our patient's ones) have been reported [3, 5, 10, 11]. The physician may detect the lesions in both sun‐exposed and sun‐protected areas of the body [7, 10].

The most frequent dermoscopic findings of PK include keratin rim, gray‐brown pigmentation, dotted or glomerular vessels, and non‐peripheral scales [21]. However, many of these findings are not reported in scalp FPK dermoscopy [3]. In our patient, the dermoscopy of scalp lesions shared some features with previous scalp FPK dermoscopy (pink areas and follicular yellow keratotic plugs). However, the buttock lesions showed unique features, like perifollicular white rim, radiating white lines, and dotted vessels.

Eosinophil exocytosis (detected in our patient) is an uncommon finding in the histopathologic assessment of FPK [2, 3]. Cornoid lamella, the hallmark of porokeratosis, was less prominent in scalp lesions compared to buttock lesions.

Some diseases were associated with the reported cases of FPK, including skin cancers (not on FPK lesions), Gilbert syndrome, Sturge–Weber syndrome, hidradenitis suppurativa, and cystic fibrosis [5, 6, 11]. Diabetes mellitus, diagnosed in our patient, was not reported in FPK. However, diabetes mellitus may accompany other types of PK [12], especially genital PK [13]. It is known that glucose changes keratinocytes' proliferation, differentiation, and migration. Therefore, high glucose levels alter the keratinocyte differentiation and proliferation balance [12, 22]. These effects may explain the associated diabetes mellitus with PK. To our knowledge, this is the first case of FPK associated with diabetes mellitus.

Topical and systemic medications, photodynamic therapy, laser therapy, cryotherapy, and surgery are suggested for PK treatment [1, 23]. Follicular porokeratosis cases have been treated with topical 5‐fluorouracil, imiquimod 5%, mometasone, and tretinoin, without satisfactory results [5, 7, 9, 11, 15, 16]. One patient received acitretin but could not tolerate it [5]. Due to the rarity of FPK, it is hard to evaluate the effectiveness of treatments. Systemic retinoids are effective treatments in other PK types [1], and oral isotretinoin caused the improvement of keratotic papules and spicules in our patient. However, isotretinoin could not change the alopecia progression in our patient.

To conclude, follicular porokeratosis should be considered among the differential diagnoses of keratotic papules (with or without spicules) and in the cases of alopecia with keratotic papules. Dermoscopy could be helpful in the diagnosis of FPK. Diabetes mellitus type 2 may be associated with FPK.

## Author Contributions

**Mitra Abdolvand:** investigation, writing – original draft, writing – review and editing. **Roya Radanfar:** investigation, writing – original draft, writing – review and editing. **Mozhdeh Sepaskhah:** conceptualization, investigation, supervision, writing – original draft, writing – review and editing. **Fatemeh Sari Aslani:** investigation, writing – review and editing. **Mojgan Akbarzadeh Jahromi:** investigation, writing – review and editing.

## Ethics Statement

The researchers committed to maintaining the patient confidentiality. Institutional ethics committee approved the case report (ethics code: IR.SUMS.MED.REC.1401.402).

## Consent

The patient signed written informed consent to permit the publication of the case report without identifying data and to use the photography for publication. The researchers committed to maintaining the patient confidentiality. Institutional ethics committee approved the case report (ethics code: IR.SUMS.MED.REC.1401.402).

## Conflicts of Interest

The authors declare no conflicts of interest.

## Data Availability

Data sharing is not applicable to this article as no new data were created or analyzed in this study.
